# Efficacy and safety of Ashwagandha root extract sustained-release (AshwaSR) capsules in healthy adult, stressed subjects: A randomized, double-blind, placebo-controlled, parallel-group, 3-arm clinical trial

**DOI:** 10.1097/MD.0000000000047990

**Published:** 2026-03-13

**Authors:** Shefali Thanawala, Rajat Shah, Krishnaraju Venkata Alluri, Kiran Bhupathiraju, Prabakaran Desomayanandanam, Arun Bhuvenendran

**Affiliations:** a Medical Science & Research, Nutriventia Private Limited, Mumbai, Maharashtra, India; b Executive Management, Nutriventia Private Limited, Mumbai, Maharashtra, India; c Pharmacology & Clinical Research, Laila Nutra Private Limited, Vijayawada, Andhra Pradesh, India; d Research & Development, Laila Nutra Private Limited, Vijayawada, Andhra Pradesh, India; e Clinical Operations, In vitro Research Solutions Pvt Ltd, Bangalore, Karnataka, India; f Statistics & Data Analytics, In vitro Research Solutions Pvt Ltd, Bangalore, Karnataka, India.

**Keywords:** ashwagandha, psychological well-being, sleep, stress, sustained-release, withanolides

## Abstract

**Background:**

The adaptogenic effects of Ashwagandha root extract are evident. An earlier study showed the therapeutic effects of a once-daily sustained-release (SR) formulation (300 mg) of Ashwagandha root extract over an extended period. This study aimed to evaluate the efficacy and safety of Ashwagandha root extract sustained-release (AshwaSR) 150 and 300 mg capsules in reducing stress in healthy adult, stressed subjects.

**Methods:**

In this double-blind, randomized, placebo-controlled trial, healthy subjects with Perceived Stress Scale score 14 to 26 were randomized (1:1:1) to AshwaSR 150 mg (group A) or 300 mg (group B) or placebo (group C). Change from baseline to day 60 was evaluated for stress levels, sleep quality, psychological well-being, eating behavior, and serum cortisol levels in all groups.

**Results:**

Of 135 subjects randomized, 126 completed the trial (mean age, 34.79 ± 8.16 years). Mean Perceived Stress Scale scores significantly reduced from baseline to day 60 in group A and B (mean change, 38.6% and 41.6% respectively; *P* < .001). Sleep quality, psychological well-being, and eating behavior significantly improved from baseline to day 60 in groups A and B (*P* < .001). Serum cortisol levels in group B were significantly reduced on day 60 (*P* < .05). Both group A and B showed significant improvements in stress levels, sleep quality, psychological well-being, and eating behavior at day 60 (*P* < .05) compared to group C. No safety concerns were reported.

**Conclusion:**

AshwaSR 150 and 300 mg capsules reduced perceived stress and improved sleep quality, eating behavior, and psychological well-being and were safe in healthy adult, stressed subjects over 60 days of administration.

## 1. Introduction

Stress is a normal response to difficult situations in an individual’s life; however, prolonged and excessive mental or physical stress can be detrimental. Chronic stress affects cognition and results in decreased functioning of multiple domains affecting daily activities which in turn can give rise to several conditions, such as depression, hypertension, and metabolic disorders.^[1,2]^ Furthermore, individuals suffering from chronic stress are vulnerable to negative emotions, such as compulsion, hostility, paranoia, anxiety, and depression, in addition to interpersonal sensitivity, sleep disruption, and altered eating patterns.^[3,4]^ The high global prevalence (~25%) and detrimental effects of stress underscore the importance of effective stress management to safeguard individuals’ mental, emotional, and physical health.^[5]^ Additionally, there exists a gap in the available approaches for effective stress management in standard medical practice. These factors together underline the requirement for effective stress management options.

Adaptogens are substances derived from certain plants that can improve an individual’s ability to cope with stress and aid homeostasis or physiological balance.^[1]^ They also have neuroprotective, anti-fatigue, antidepressant, anxiolytic, nootropic, and central nervous system stimulating properties.^[6]^ Ashwagandha, *Withania somnifera* (known as Indian ginseng or winter cherry), is an Indian herb which has been used in Ayurveda, the traditional Indian medicine, as an adaptogen since approximately 3000 BC.^[7]^ Ashwagandha root extract has been demonstrated to augment the immune system, promote healthy aging, rejuvenate the body systems, boost resistance to adverse environmental factors, and promote a sense of well-being.^[8]^ The pharmacologically active phytochemical constituents of Ashwagandha root are steroidal lactones and their glycosides, known as withanolides.^[9]^

Clinical studies have demonstrated the efficacy of withanolides in reducing chronic stress, anxiety, and cortisol levels, suggesting their potential role in the management of stress.^[1,9]^ Ashwagandha root powder is required to be administered in grams divided over 2 to 3 servings daily, and standard immediate-release Ashwagandha formulations are commonly prescribed in clinical practice in a twice- or thrice- daily dose. Ashwagandha root extract sustained-release (SR) capsule 300 mg, a formulation developed to provide convenience of administration and improve compliance, and previously evaluated in our earlier published study, was used in the present study.^[10]^ The sustained-release formulation technology of AshwaSR allows the Ashwagandha root extract to exert therapeutic effects for an extended duration with a single daily dose. This, in turn, enhances convenience and compliance by reducing the dosing frequency. The SR formulation showed superior bioavailability of withanolides and a longer elimination half-life indicating a SR profile after single daily administration compared to the reference product (marketed Ashwagandha root extract capsule).^[10,11]^ The efficacy of AshwaSR capsules (Prolanza™/Ashwanova™, Nutriventia Private Limited, Mumbai, Maharashtra, India, and Laila Nutra Private Limited, Vijayawada, Andhra Pradesh, India) was demonstrated in preclinical and clinical studies.^[11,12]^ In an in vitro study, the dose-dependent anti-neuroinflammatory activity of AshwaSR was shown in terms of inhibition of tumor necrosis factor alpha, interleukin-1 beta, and superoxide production. Additionally, in an in vivo assessment of the chronic unpredictable stress model, AshwaSR demonstrated anxiolytic and stress-relieving effects.^[11]^ Further, a randomized, double-blind, placebo-controlled clinical study demonstrated improvement in cognitive function, psychological well-being, sleep quality, and stress level in healthy adult, stressed subjects after treatment for 90 days with AshwaSR 300 mg in a single daily dose compared to placebo.^[12]^

There is a paucity of evidence assessing the efficacy and safety of low-dose formulations of Ashwagandha in stress management. This study aimed to assess whether a low-dose formulation (150 mg) of AshwaSR capsules could effectively reduce stress compared to its standard-dose SR formulation (300 mg) or placebo. We evaluated the efficacy and safety of AshwaSR capsules at doses of 150 and 300 mg in reducing stress and associated symptoms in healthy adults experiencing stress, over a period of 60 days of administration. Additionally, we evaluated the effects of both doses of AshwaSR on mood, sleep quality, impaired eating habits due to stress, and serum cortisol levels.

## 2. Materials and methods

### 2.1. Study design

This was a 60-day randomized, double-blind, 3-arm, parallel-group, placebo-controlled prospective clinical trial with recruitment completed between 15th June 2023 and 23rd October 2023 at 2 sites (Bangalore Neuro Centre, Bangalore and Santhosh Hospital, Bangalore) in India.

### 2.2. Ethical considerations

The study was conducted in accordance with the Good Clinical Practice and the Helsinki Declaration Standards, in addition to The New Drug and Clinical Trial Rules 2019 and the Ethical Guidelines for Biomedical Research on Human Subjects, issued by the Indian Council of Medical Research. The study was registered on Clinical Trials Registry-India (CTRI) on 08/06/2023 (CTRI Reg no: CTRI/2023/06/053662). The study protocol, informed consent form and other relevant study documents were reviewed and approved by an independent, registered Santhosh Hospital – Institutional Ethics Committee (Reg. no. ECR/1062/Inst/KA/2018/RR-21) before initiating the study recruitment. A written informed consent was obtained from each subject prior to study commencement.

### 2.3. Key eligibility criteria of subjects

The study included healthy male or female subjects, aged 20 to 55 years, with a body mass index of 18 to 30 kg/m^2^ and a Perceived Stress Scale (PSS) score between 14 and 26. Female subjects of childbearing potential were required to use a medically acceptable form of birth control other than contraceptives, and those of non-childbearing potential needed to be amenorrheic for ≥1 year or have undergone hysterectomy and/or bilateral oophorectomy.

Subjects with psychiatric illnesses as per the Diagnostic and Statistical Manual of Mental Disorders, 5th edition criteria; uncontrolled systemic illnesses; concurrent use of supplements for stress, anxiety, mood, and sleep or other indications; concurrent use of β-blockers or contraceptive or psychotropic medications; who were treated for infertility or sexual function enhancement within 6 months prior to study enrollment; use of body composition enhancing agents; who demonstrated hypersensitivity to any of the ingredients in the study products; alcohol dependence or established substance abuse concerns, were excluded.

### 2.4. Randomization and treatment allocation

After screening, eligible subjects were randomized (1:1:1) to receive AshwaSR 150 mg capsules (group A), 300 mg capsules (group B) or placebo capsules (group C), according to a software-generated randomization schedule (block size of 6). The randomization schedule was generated by a study statistician using SAS^®^, version 9.4 (SAS Institute Inc., Cary). Subjects were assigned a unique randomization code by an unblinded pharmacist at each study site. Since the study was planned as a double-blind, treatment assignment was not disclosed to the subjects or investigators. In addition, the batch numbers of all investigational product containers were masked and assigned sequential numbers.

AshwaSR (Prolanza™/Ashwanova™ , Nutriventia Private Limited, Mumbai, Maharashtra, India, and Laila Nutra Private Limited, Vijayawada, Andhra Pradesh, India) is a capsule containing SR *Withania somnifera* root extract (standardized to contain not <4% of total withanolides by HPLC modified USP method) in doses of 150 or 300 mg and permitted excipients. The SR profile was tested using the in vitro dissolution test and sustained release of the phytoactives from the test product, that is, withanolides, has been demonstrated in the pharmacokinetic study conducted earlier.^[10]^ The stability of the AshwaSR capsules was assessed and the shelf-life was determined to be 2 years. Placebo capsules were manufactured to be identical to the test capsules in terms of color, size, and shape, without the active ingredient. The quality control tests performed on the study products included evaluation of weight variation, net content, disintegration test, heavy metal analysis, microbiological analysis, and residual solvents, whereas total withanolides content evaluated only for the test product. The subjects were instructed to consume 1 capsule orally with water, daily after breakfast in the morning for 60 days. A 60-day study duration was selected based on the previously published studies demonstrating stress-reducing effects of Ashwagandha root extract after 60 days of supplementation.^[1,13,14]^

### 2.5. Study procedure

The study included a total of 8 visits: screening (visit 1, day −7 to day 0), baseline/randomization (visit 2, day 1), interim visits (visits 3–7, days 3 [±1], 7 [±2], 15 [±2], 30 [±2], and 45 [±3]), and end of treatment (visit 8, day 60 [±3]). During the screening visit, the subjects were assessed for eligibility. At the baseline/randomization visit, eligible subjects were randomized and allocated to treatment. Subjects were assessed for study outcomes at baseline, interim, and end of treatment visits. The subjects’ diaries were collected and reviewed for treatment compliance during all follow-up visits. Operational measures such as standardized training of the site personnel and regular monitoring at each study site were implemented to minimize inter-site variability.

### 2.6. Study endpoints

The primary efficacy endpoint was to evaluate the change in PSS score from screening to visit 8 (day 60) in groups A and B as compared to group C.

Secondary efficacy endpoints included comparison of change in PSS score from screening to days 3, 7, 15, 30, 45, and 60 between all groups; comparison of Pittsburgh Sleep Quality Index (PSQI); Oxford Happiness Questionnaire-8 (OHQ-8) score from baseline to days 3, 7, 15, 30, 45, and 60; Three-Factor Eating Questionnaire-Revised 18 (TFEQ-R18) score; and serum cortisol level from baseline to days 7, 30, and 60 between all 3 groups. In addition, the subjects’ and physicians’ global assessment of therapy at the end of treatment was assessed as a secondary efficacy endpoint.

Treatment-emergent adverse events (AEs) were reported under safety assessment.

### 2.7. Study assessment

The PSS score^[15]^ was assessed using a 10-item subjective questionnaire, which is the most widely used psychological instrument for measuring the perception of stress in terms of the degree to which situations in an individual’s life are appraised as stressful. Items were designed to determine how unpredictable, uncontrollable, and overloaded subjects found their lives, with response scores ranging between 0 and 40 (scores 0–13, low; 14–26, moderate; 27–40, high perceived stress).^[16]^

The PSQI^[17]^ is an effective instrument for measuring the quality and patterns of sleep. It differentiates “poor” from “good” sleep by measuring 7 domains: subjective sleep quality, sleep latency, sleep duration, habitual sleep efficiency, sleep disturbances, use of sleep medication, and daytime dysfunction. Responses to each of the 7 domains were measured on a Likert Scale of 0 to 3, giving a global PSQI score of 0 to 21. A global PSQI score of more than 5 indicated poor sleep relative to clinical and laboratory measures.^[18]^

The TFEQ-R18^[19,20]^ consists of 18 items on a 4-point Likert scale (1 = definitely true, 2 = mostly true, 3 = mostly false, 4 = definitely false). Responses to each of the 18 items are summated into scale scores for cognitive restraint (6-item), uncontrolled eating (9-item), and emotional eating (3-item). Higher scores in the respective scales are indicative of greater cognitive restraint, and uncontrolled or emotional eating.

The OHQ-8^[21]^ consists of 8 items with a series of statements to which respondents indicate their agreement or disagreement on a scale ranging from 1 = strongly disagree, 2 = moderately disagree, 3 = slightly disagree, 4 = slightly agree, 5 = moderately agree, and 6 = strongly agree. It is a widely used psychological assessment tool designed to measure an individual’s subjective psychological well-being. A score of 4 or above indicates enhanced psychological well-being in subjects.^[22]^

For serum cortisol estimation, blood samples were collected from the subjects in the morning (9–11 am).

This trial was powered specifically to detect a clinically meaningful difference in the PSS score which was the primary endpoint. Accordingly, the sample size determination and statistical analyses were centered on changes in the PSS score. All other outcomes (including sleep quality, happiness, eating behavior, cortisol, and global assessments) were analyzed as secondary endpoints; each secondary outcome was evaluated independently to explore different domains of interest and to provide a broader perspective on the potential effects of the intervention.

### 2.8. Statistical analysis

Sample size was calculated based on a previous double-blind, randomized, parallel-group, placebo-controlled study^[12]^ where the true difference between the test and placebo in terms of PSS was 5.7 (13.0 and 18.7 for the test and placebo products, respectively, at the end of treatment) and the expected population standard deviation (SD) was assumed to be 8.2. To establish superiority between the test and placebo at 80% power at a 5% level of significance (i.e., *α* = .05) with equal allocation (i.e., *k* = 1) and approximately 15% drop out and noncompliance, the sample size of 45 subjects per group was obtained. Therefore, a total of 135 subjects were enrolled and equally randomized to the test and placebo arms, to achieve evaluable subjects totaling 38 per arm (total 114 subjects to complete the trial).

A treatment compliance of ≥80% was considered acceptable for efficacy analysis. Subjects who completed the study treatment without any major protocol deviations with treatment compliance of at least ≥80% were included in the per-protocol population analysis.

Statistical analysis was performed using SAS^®^ version 9.4. Descriptive statistics were used for qualitative data (presented as number and percentage) and quantitative data (presented as mean and SD). Homogeneity of demographic data pertaining to each treatment and baseline values of each outcome measure were verified using analysis of variance. To evaluate variance among all study treatment arms, analysis of variance for independent means was used. This was followed by post hoc Tukey honest significant difference to evaluate the honest significant difference among paired comparisons of study treatment arms. Covariate adjustment with baseline values as covariates was carried out for all parameters to nullify the influence of baseline differences among the parameters. In addition, Student *t*-test for independent measures was used, as applicable, to evaluate differences between active study treatment arms. A *P* value < .05 was considered statistically significant.

## 3. Results

### 3.1. Subject disposition and characteristics

**Figure 1.**
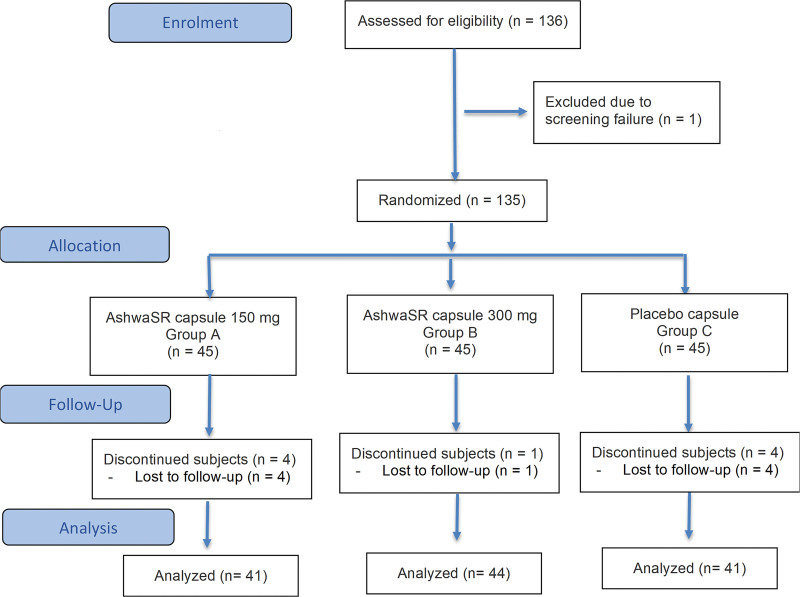
CONSORT flow diagram. AshwaSR = Ashwagandha root extract sustained-release, CONSORT = consolidated standards of reporting trials.

Of the 136 subjects screened, 135 were randomized (screen failure, n = 1) to group A, group B, or group C. Of these, 9 subjects were lost to follow-up (group A [n = 04]; group B [n = 01]; and group C [n = 04]) by the end of the study. Overall, 126 subjects completed the study with treatment compliance of ≥80%, and were included in the analysis (group A, n = 41; group B, n = 44; group C, n = 41; Fig. 1). At baseline, age (mean ± SD) of subjects was 34.79 ± 8.16 years, and 53.17% were male. Demographics and baseline characteristics of subjects are summarized in Table 1. The subjects did not differ in demographics or baseline characteristics across arms (*P* > .05).

**Table 1:** Demographic details and baseline characteristics.

Parameters	Group A AshwaSR 150 mg (n = 41)	Group B AshwaSR 300 mg (n = 44)	Group C Placebo (n = 41)	*P* value
Age (yr)	34.29 ± 7.77	34.61 ± 8.97	35.49 ± 7.77	.792
Body weight (kg)	67.28 ± 10.58	64.65 ± 9.21	65.55 ± 8.57	.435
Height (cm)	164.10 ± 8.29	163.47 ± 7.83	162.18 ± 7.71	.458
BMI (kg/m^2^)	24.91 ± 2.85	24.17 ± 2.75	24.89 ± 2.37	.309

Data are presented as mean ± SD.

*P* value derived from ANOVA for independent means followed by post hoc Tukey HSD.

ANOVA = analysis of variation, AshwaSR = Ashwagandha root extract sustained-release, BMI = body mass index, HSD = honest significant difference, SD = standard deviation.

### 3.2. Primary outcomes

#### 3.2.1. Change in PSS score

**Figure 2.**
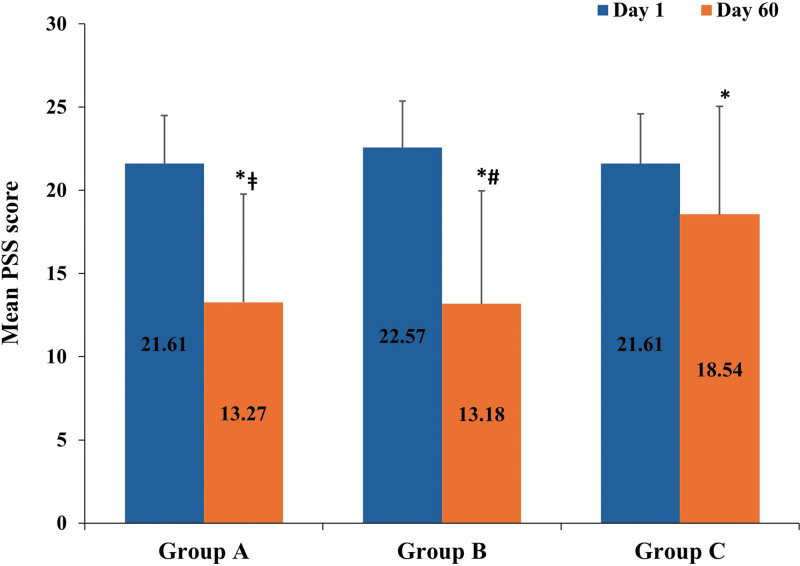
Perceived Stress Scale score at baseline and day 60. ^*^Shows *P* value within treatment, comparison between baseline/day 1 and day 60 (*P* value derived from *t*-test for dependent measures; *P* < .05 indicates statistically significant difference). ^ǂ^Shows *P* value for comparison between group A and group C on respective days (*P* value derived from post hoc Tukey HSD; *P* < .05 indicates statistically significant difference). ^#^Shows *P* value for comparison between group B and group C on respective days (*P* value derived from post hoc Tukey HSD; *P* < .05 indicates statistically significant difference). Error bars represent standard deviation. Number of subjects included in group A, group B and group C were 41, 44, and 41, respectively. Group A: AshwaSR 150 mg capsules; group B: AshwaSR 300 mg capsules; group C: placebo. AshwaSR = Ashwagandha root extract sustained-release, HSD = honest significant difference, PSS = Perceived Stress Scale.

The PSS score (mean ± SD) in group A, group B, and group C was 21.61 ± 2.89, 22.57 ± 2.79, and 21.61 ± 2.99 at baseline, respectively. The mean PSS score at baseline was not significantly different between the study groups (*P* > .05). The PSS score for group A, group B, and group C significantly reduced from baseline to 13.27 ± 6.51, 13.18 ± 6.79, and 18.54 ± 6.51 at day 60, respectively (*P* < .05). Mean change in PSS score (%) from baseline to day 60 was higher in group B (41.6%), followed by group A (38.6%) and group C (14.2%; Fig. 2). Compared to baseline, the mean PSS score significantly reduced as early as day 15 in group A and group B, and reduction was sustained up to day 60 (all *P* < .05). Compared to group C, the mean PSS score significantly reduced as early as day 15 in group B and day 30 in group A. The reduction was sustained up to day 60 (all *P* < .05). Mean PSS score was comparable between group A and group B at all visits (Table 2).

**Table 2:** Perceived Stress Scale, Pittsburgh Sleep Quality Index, and Oxford Happiness Questionnaire-8 score from baseline to end of treatment.

Items	Day 1	Day 3	Day 7	Day 15	Day 30	Day 45	Day 60
PSS score							
Group A	21.61 ± 2.89	20.85 ± 4.60	20.71 ± 4.91	19.63 ± 5.05*	17.76 ± 4.32*†	16.90 ± 5.21*†	13.27 ± 6.51*†
Group B	22.57 ± 2.79	22.93 ± 3.54	22.11 ± 2.97	20.02 ± 3.59*§	18.20 ± 3.64*§	16.75 ± 5.34*§	13.18 ± 6.79*§
Group C	21.61 ± 2.99	21.76 ± 3.65	21.95 ± 4.22	21.37 ± 3.85	21.10 ± 3.92	19.78 ± 4.31*	18.54 ± 6.51*
PSQI global score							
Group A	9.44 ± 3.35	9.15 ± 3.68	8.32 ± 3.43*†	7.56 ± 3.35*†	7.07 ± 3.58*†	6.32 ± 3.14*†	5.54 ± 3.02*†
Group B	9.32 ± 3.34	8.82 ± 3.69*	8.07 ± 3.54*§	7.20 ± 3.72*§	6.25 ± 3.17*§	5.16 ± 3.09*§	4.52 ± 2.90*§
Group C	8.17 ± 3.26	8.17 ± 2.92	8.15 ± 2.83	8.20 ± 2.89	7.98 ± 3.00	7.80 ± 3.27	7.59 ± 3.46
OHQ-8 score							
Group A	3.14 ± 0.52†	3.26 ± 0.67	3.37 ± 0.62*	3.44 ± 0.50*†	3.46 ± 0.55*	3.63 ± 0.58*†	3.75 ± 0.60*
Group B	2.93 ± 0.60§	2.90 ± 0.60	3.19 ± 0.66*§	3.32 ± 0.62*§	3.47 ± 0.56*§	3.72 ± 0.54*§	4.00 ± 0.65*§‡
Group C	3.47 ± 0.48	3.47 ± 0.36	3.49 ± 0.38	3.51 ± 0.41	3.55 ± 0.45	3.64 ± 0.50*	3.73 ± 0.60*

Data are presented as mean ± SD.

No. of subjects included in group A, group B and group C were 41, 44, and 41, respectively.

Group A: AshwaSR 150 mg capsules; group B: AshwaSR 300 mg capsules; group C: placebo.

AshwaSR = Ashwagandha root extract sustained-release, HSD = honest significant difference, OHQ-8 = Oxford Happiness Questionnaire-8, PSQI = Pittsburgh Sleep Quality Index, PSS = Perceived Stress Scale, SD = standard deviation.

* Shows *P* value within treatment, compared to baseline/day 1 (*P* value derived from *t*-test for dependent measures; *P* < .05 indicates statistically significant difference).

† Shows *P* value for comparison between group A and group C on respective days (*P* value derived from post hoc Tukey HSD; *P* < .05 indicates statistically significant difference).

‡ Shows *P* value for comparison between group A vs group B on respective days (*P* value derived from post hoc Tukey HSD; *P* <.05 indicates statistically significant difference).

§ Shows *P* value for comparison between group B and group C on respective days (*P* value derived from post hoc Tukey HSD; *P* <.05 indicates statistically significant difference).

### 3.3. Secondary outcomes

#### 3.3.1. Change in PSQI global score

At baseline, PSQI global score (mean ± SD) in group A, group B, and group C was 9.44 ± 3.35, 9.32 ± 3.34, and 8.17 ± 3.26, respectively, and was comparable (*P* > .05). At day 60, the PSQI global score (mean ± SD) significantly reduced from baseline to 5.54 ± 3.02 in group A and 4.52 ± 2.90 in group B (*P* < .05), with a higher mean change (%) from baseline observed in group B (51.5%), followed by group A (41.3%). Compared to baseline, the PSQI global score significantly reduced as early as day 3 in group B and day 7 in group A (*P* < .05), and the reduction was sustained up to day 60; the PSQI global score in group C was 7.59 ± 3.46 at day 60 (mean change (%) from baseline 6.6%, *P* = .178). Moreover, PSQI score was not significantly different from baseline until day 60 in group C. Compared to group C, PSQI global score significantly reduced as early as day 7 in group A and group B (Table 2).

#### 3.3.2. Change in OHQ-8 score

The OHQ-8 score was 3.14 ± 0.52 in group A, 2.93 ± 0.6 in group B, and 3.47 ± 0.48 in group C at baseline. At day 60, the OHQ-8 score increased significantly to 3.75 ± 0.60 in group A, 4.00 ± 0.65 in group B, and 3.73 ± 0.60 in group C (*P* < .05), with a higher mean change from baseline observed in group B (36.3%), followed by group A (19.2%), and group C (7.6%). Compared to baseline, OHQ-8 score significantly improved as early as day 7 in group A and group B (*P* < .05), and the improvement was sustained up to day 60. Compared to group C, mean OHQ-8 score significantly improved as early as day 7 until day 60 in group B, whereas the OHQ-8 score significantly improved as early as day 15 in group A *vs* group C. The improvement was sustained until day 60; however, it was not significant over group C. Furthermore, the mean OHQ-8 score was comparable between group A and group B at all visits except day 60 (Table 2).

#### 3.3.3. Change in TFEQ-R18 score

The TFEQ-R18 score for cognitive restraint, uncontrolled eating, and emotional eating is summarized in Table 3. Compared to baseline, the TFEQ-R18 score for cognitive restraint significantly reduced to 10.59 ± 2.77 in group A and 10.93 ± 3.41 in group B at day 60 (*P* < .05), with a slightly higher mean change (%) of 22.78% observed in group A followed by 21.53% in group B. Similarly, TFEQ-R18 score for uncontrolled eating and emotional eating significantly reduced in group A (14.95 ± 3.71 and 4.95 ± 1.53) and group B (14.25 ± 3.50 and 4.73 ± 1.34), respectively, at day 60 (*P* < .05), with a higher mean change (%) in TFEQ-R18 score for uncontrolled eating and emotional eating observed in group B (23.91% and 24.64%), followed by group A (22.11% and 22.22%), respectively. Compared to baseline, the TFEQ-R18 scores for cognitive restraint and uncontrolled eating were significantly reduced at day 7 up to day 60, and the score for emotional eating was significantly reduced at days 30 and 60 in group A and group B (all *P* < .05). There was no significant reduction in scores from baseline to day 60 in group C. Compared to group C, TFEQ-R18 score for cognitive restraint was significantly reduced at days 30 and 60 in group A and group B (*P* < .05), while the TFEQ-R18 score for uncontrolled eating was significantly reduced as early as day 7 up to day 60 in group A and group B. Furthermore, TFEQ-R18 scores for emotional eating were significantly reduced at day 60 in group A and group B compared to group C. The TFEQ-R18 score for cognitive restraint, uncontrolled eating, and emotional eating was comparable between group A and group B at all visits.

**Table 3:** Three-Factor Eating Questionnaire-Revised 18 score from baseline to end of study.

Items	Day 1	Day 7	Day 30	Day 60
TFEQ-R18 - cognitive restraint score				
Group A	13.71 ± 2.96	12.41 ± 2.31*	12.02 ± 2.57*†	10.59 ± 2.77*†
Group B	13.93 ± 2.64	12.82 ± 2.69*	11.75 ± 3.05*‡	10.93 ± 3.41*‡
Group C	13.00 ± 2.39	12.56 ± 2.52	12.63 ± 2.80	12.17 ± 3.24
TFEQ-R18 - uncontrolled eating score				
Group A	19.20 ± 4.44	17.66 ± 3.95*†	16.66 ± 3.81*†	14.95 ± 3.71*†
Group B	18.73 ± 4.76	17.32 ± 4.34*‡	15.77 ± 3.55*‡	14.25 ± 3.50*‡
Group C	17.93 ± 3.88	17.95 ± 4.08	18.05 ± 4.40	17.54 ± 5.19
TFEQ-R18 - emotional eating score				
Group A	6.37 ± 1.96	6.20 ± 1.74	5.59 ± 1.41*	4.95 ± 1.53*†
Group B	6.27 ± 1.47	6.07 ± 1.50	5.43 ± 1.28*	4.73 ± 1.34*‡
Group C	6.15 ± 1.73	6.08 ± 1.38	6.02 ± 1.75	6.02 ± 1.89

Data are presented as mean ± SD.

No. of subjects included in group A, group B and group C were 41, 44, and 41, respectively.

Group A: AshwaSR 150 mg capsules; group B: AshwaSR 300 mg capsules; group C: placebo.

AshwaSR = Ashwagandha root extract sustained-release, HSD = honest significant difference, SD = standard deviation, TFEQ-R18 = Three-Factor Eating Questionnaire-Revised 18.

* Shows *P* value within treatment, compared to baseline/day 1 (*P* value derived from *t*-test for dependent measures; *P* < .05 indicates statistically significant difference).

† Shows *P* value for comparison between group A and group C on respective days (*P* value derived from post hoc Tukey HSD; *P* < .05 indicates statistically significant difference).

‡ Shows *P* value for comparison between group B vs group C on respective days (*P* value derived from post hoc Tukey HSD; *P* < .05 indicates statistically significant difference).

#### 3.3.4. Serum cortisol level

Compared to baseline, at day 60 serum cortisol levels (mg/dL) in group B were reduced significantly (7.83 ± 2.61 vs 6.33 ± 2.01; *P* < .05); whereas, these were reduced numerically in group A (7.96 ± 2.88 vs 7.05 ± 2.46; *P* = .212) and group C (7.05 ± 2.74 vs 6.63 ± 2.17; *P* = .562).

#### 3.3.5. Subjects’ and physicians’ global assessment of therapy

The majority of subjects reported treatments as “Good/Very Good” (group A, 95.12%; group B, 84.09%; group C, 90.24%). Likewise, most physicians reported treatments as “Good/Very Good” (group A, 97.56%; group B, 97.73%; group C, 92.68%).

#### 3.3.6. Safety

Overall, 5 subjects reported self-limiting, mild, and transient AEs such as pyrexia, myalgia, emesis, pruritus, and gastroesophageal reflux during the study period (group A, n = 3; group B, n = 1; group C, n = 1). Duration of AEs was limited to 4 to 5 days, and as per the causality assessment, the AEs were unrelated to the study treatment and completely resolved without any complications, allowing all subjects to continue their participation in the study. The AEs were comparable in terms of duration, severity, and seriousness in group A and group B to those observed in group C.

## 4. Discussion

This study assessed the efficacy and safety of AshwaSR 150 and 300 mg capsules in a single daily dose in healthy adult, stressed subjects. The results showed that AshwaSR 150 and 300 mg capsules improved perceived stress level, sleep quality, eating behavior, and overall well-being in subjects over 60 days of treatment. Improvements in outcomes (from baseline to day 60) were comparable between 150 and 300 mg SR capsules; however, a quicker and higher percentage of improvement was observed with AshwaSR 300 mg capsules. Furthermore, AshwaSR 300 mg capsules demonstrated reduction in perceived stress level as early as day 15 and improvement in psychological well-being and uncontrolled eating as early as day 7 after administration, which lasted over 60 days of treatment.

Stress, whether from internal or external sources, acts as a triggering or aggravating factor for many diseases and pathological conditions. Therefore, it is crucial to prevent and manage stress effectively.^[23]^ In this study, PSS score was reduced from 22.57 at baseline to 13.18 at day 60 in the AshwaSR 300 mg-treated group, which was similar to reductions reported in previous clinical studies on Ashwagandha extract^[1,12]^; whereas, in the first clinical trial of AshwaSR 300 mg capsules, a study by Gopukumar et al, PSS score was reduced from 19.5 to 13.0 at day 90 with AshwaSR 300 mg.^[12]^ A double-blind, randomized, placebo-controlled trial showed reduction in PSS score from 22.95 to 14.15 after 8 weeks of treatment with Ashwagandha root extract 600 mg in healthy adults.^[13]^ In this study, PSS score was reduced by 41.6% with 300 mg and 38.6% with AshwaSR 150 mg capsules, which was close to the reductions observed after 60 days of treatment with Ashwagandha 300 mg capsules in the previous study.^[1]^ While a degree of improvement in stress scores was seen with placebo, possibly due to psychological and contextual factors, the magnitude of reduction observed with Prolanza™ was nearly 3-fold higher than placebo, suggesting a robust treatment effect. This could be attributed to anti-neuroinflammatory activity of Ashwagandha root extract, as demonstrated in terms of dose-dependent inhibition of tumor necrosis factor alpha, interleukin-1 beta, and superoxide production in an in vitro study.^[11]^

Sleep is considered an important physiological phenomenon, with a restorative and rejuvenating influence on physiological systems,^[24]^ which can be disrupted by elevated stress levels. Disturbed sleep and poor sleep quality compromise cognitive function, mood regulation, overall well-being, and happiness.^[25]^ A randomized, double-blind, placebo-controlled study in healthy subjects showed improved sleep quality after 6 weeks of treatment with Ashwagandha root extract (120 mg); in particular in subjects with frequent non-restorative sleep.^[26]^ Our study demonstrated a significant improvement in PSQI global score within 60 days of treatment with AshwaSR 300 and 150 mg capsules. Moreover, improvement in sleep quality was observed from as early as day 3 with AshwaSR 300 mg and day 7 with AshwaSR 150 mg capsules. The improvement in sleep quality may be attributed to an adaptogenic effect of Ashwagandha to regulate mood and overall well-being.^[8]^

Furthermore, a PSQI score < 5 indicates good sleep quality^[18]^ and an OHQ-8 score ≥ 4 indicates enhanced psychological well-being in subjects.^[22]^ In this study, PSQI and OHQ-8 scores of 4.52 and 4.00, respectively, at day 60 demonstrated subjects experienced good sleep quality and enhanced psychological well-being with AshwaSR 300 mg. In contrast, PSQI and OHQ-8 scores of 5.54 and 3.75, respectively, in subjects with AshwaSR 150 mg did not surpass respective thresholds at day 60. However, the trend of improvement in scores suggests that treatment with AshwaSR 150 mg for an increased time period may be required to cross cutoff values.

Perceived stress has been demonstrated to have a significantly negative impact on eating behavior.^[27]^ A higher cognitive restraint can lead to inhibition which is frequently interspersed with loss of control, described as hyperphagic or bulimic bouts and compulsive eating. A more rational strategy to normalize the eating behavior is probably to restore the individual’s hunger and satiety physiological regulation systems.^[28]^ In this study, AshwaSR 300 and 150 mg capsules demonstrated a restoration of positive eating behavior with an optimum balance of all the 3 domains of eating behavior as evaluated by the TFEQ-R18. A reduction in the cognitive restraint and uncontrolled eating scores from day 7 was observed with reduction in emotional eating score from day 30, which lasted till day 60 of the treatment.

Cortisol plays a crucial role in regulating the body’s stress response from onset to recovery. Variation in cortisol level has been observed during instances of psychological stress.^[29]^ Ashwagandha root extract, by acting on the hypothalamic–pituitary–adrenal axis, normalizes cortisol secretion and prevents excessive elevation of cortisol during stress.^[29,30]^ Several clinical studies have reported reduction in cortisol level after treatment with Ashwagandha root extract.^[1,13,31]^ Similarly, in this study, serum cortisol levels reduced from baseline to day 60 by 19.15% with 300 mg and by 11.44% with 150 mg AshwaSR, which suggests the association of the stress-reducing ability of Ashwagandha root extract with reduction in serum cortisol levels. While AshwaSR 300 mg showed significant reduction on day 60 from baseline, the AshwaSR 150 mg group also showed a trend of decreasing cortisol levels over the study duration. This suggests that AshwaSR 150 mg reduces cortisol levels gradually and the reduction may reach statistical significance if administered for a longer duration.

AshwaSR 150 and 300 mg capsules were well tolerated, without any serious AEs reported throughout treatment. In this study, AEs were mild and did not result in subjects withdrawing from the study.

To our knowledge, this was a first-of-its-kind randomized 3-arm, placebo-controlled, multicenter study that compared a low-dose formulation of AshwaSR with a standard-dose AshwaSR capsules (150 vs 300 mg) and demonstrated multiple benefits in terms of improvement in stress, sleep quality, eating behavior, and overall well-being in healthy adult subjects with stress, over a period of 60 days of treatment. However, with a daily dose of AshwaSR 150 mg, administration for an increased time period may be required for achieving the threshold for good sleep quality score (PSQI). Moreover, AshwaSR 300 mg capsules showed a reduction in perceived stress levels that was observed as early as day 15, with improvement in psychological well-being and eating behavior as early as day 7 of administration, and these effects were sustained until the end of treatment (60 days). In addition, the study reported the effect of AshwaSR 300 and 150 mg capsules on serum cortisol reduction over 60 days. All these demonstrated positive effects of the test product are also contributed by the stringent and consistent analytical and quality standards employed in the manufacturing process, which are essential for ensuring reproducible efficacy.^[32]^

Limitations of the study should be considered. The effects of AshwaSR 300 and 150 mg capsules were evaluated over 60 days; however, long-term follow-up could provide further insights into safety aspects. Also, further studies in older subjects could help to provide insights into efficacy across diverse age groups. Perceived stress was assessed using the validated PSS-10 questionnaire; however, specific stressors and interim lifestyle or environmental changes or stressful events were not tracked during the study period and may be explored in future studies. Lastly, results of global assessment of therapy should be considered supportive rather than confirmatory, in conjunction with the other endpoints.

## 5. Conclusion

AshwaSR capsules at doses of 150 and 300 mg showed multiple benefits, in terms of improvement in perceived stress level, improvement of sleep quality, reinforcement of positive eating behavior, and enhancement of psychological well-being in healthy adult, stressed subjects. Compared to the low-dose AshwaSR (150 mg), treatment with standard-dose AshwaSR (300 mg) capsules demonstrated a rapid and sustained improvement in stress levels, psychological well-being, and eating behavior over 60 days of study duration suggesting dose-dependent differences in achieving these benefits, indicating that studies with longer duration for lower dose may be planned for achieving similar effects. Both doses of AshwaSR (150 and 300 mg) capsules were well tolerated and safe over 60 days. Overall, the study demonstrated beneficial effects of AshwaSR 150 and 300 mg capsules in the management of stress.

## Acknowledgments

We would like to thank Roshni Patel, PhD and Sonali Dalwadi, PhD, CMPP™ of IWANA Consultancy Solutions for providing medical writing support for this manuscript, which was funded by Nutriventia Private Limited, Mumbai, India and Laila Nutra Private Limited.

## Author contributions

**Conceptualization:** Shefali Thanawala, Rajat Shah.

**Data curation:** Shefali Thanawala, Krishnaraju Venkata Alluri, Prabakaran Desomayanandanam, Arun Bhuvenendran.

**Formal analysis:** Shefali Thanawala, Krishnaraju Venkata Alluri, Prabakaran Desomayanandanam, Arun Bhuvenendran.

**Funding acquisition:** Rajat Shah, Kiran Bhupathiraju.

**Investigation:** Prabakaran Desomayanandanam, Arun Bhuvenendran.

**Project administration:** Shefali Thanawala.

**Resources:** Rajat Shah, Kiran Bhupathiraju.

**Supervision:** Shefali Thanawala, Krishnaraju Venkata Alluri.

**Visualization:** Rajat Shah.

**Writing – original draft:** Shefali Thanawala.

**Writing – review & editing:** Shefali Thanawala, Rajat Shah, Krishnaraju Venkata Alluri, Kiran Bhupathiraju, Prabakaran Desomayanandanam, Arun Bhuvenendra.
